# Portrayals of Depression on TikTok: Content Analysis of Diagnostic Accuracy, Creator Type, and Stylistic Features

**DOI:** 10.2196/85323

**Published:** 2026-04-13

**Authors:** Elena Rainer, Amber van der Wal, Ine Beyens

**Affiliations:** 1 Amsterdam School of Communication Research, University of Amsterdam, P.O. Box 15791, Amsterdam, NH, 1001 NG, The Netherlands, 31 205253680

**Keywords:** TikTok, mental health, depression, diagnostic accuracy, stylistic features, user engagement

## Abstract

**Background:**

Youths are increasingly turning to TikTok for mental health information, making the platform an important space where young people encounter portrayals of mental illness. While such visibility can raise awareness, reduce stigma, and make young people feel more connected and understood in their experiences, concerns have been raised about the diagnostic accuracy of this content, which is often produced by nonprofessionals and presented using emotionally appealing stylistic features. Although prior research has examined mental health content on TikTok broadly, little is known about how depression-related symptoms are portrayed by creators on the platform.

**Objective:**

Given depression’s rising prevalence among youth and its prominent presence on TikTok, this study examined (1) the diagnostic accuracy of TikTok videos about depression, (2) differences in diagnostic accuracy and stylistic features by creator type (medical professionals vs nonprofessionals), and (3) how diagnostic accuracy, stylistic features (personal experiences, emotional appeals, and background music), and creator type relate to user engagement.

**Methods:**

A quantitative content analysis was conducted of 210 English-language TikTok videos retrieved using symptom-focused search terms (eg, “depression symptoms”). Videos were coded for diagnostic accuracy using a standardized coding scheme based on the *International Classification of Diseases, 11th Revision* diagnostic criteria for depressive episodes. In addition, videos were coded for creator type, presentation style, and the presence of emotionally appealing stylistic features. Engagement was operationalized as the sum of a video’s likes, comments, saves, and shares. Intercoder reliability was assessed using Krippendorff α, percent agreement, and Gwet AC1 (agreement coefficient 1). Analyses included Mann-Whitney *U* tests, chi-square tests, and hierarchical regression.

**Results:**

Diagnostic accuracy was low overall (mean score 1.21, SD 1.04, on a 0‐4 scale) and did not differ significantly between medical professionals and nonprofessionals (median 1.40 [IQR 1-2] vs 1.11 [IQR 0-2]; *P*=.06). Hierarchical regression analysis showed that diagnostic accuracy did not predict engagement (*B*=−0.10; *P*=.19). In contrast, engagement was higher for videos containing personal experiences (*B*=0.41; *P*=.02), emotional appeals (*B*=0.73; *P*=.001), and background music (*B*=0.54; *P*=.01). Across regression models, direct-to-camera formats (*B*s −0.49 to −0.69; .003≤*P*≤.04) and text-centered videos (*B*s −0.56 to −0.64; .002≤*P*<.01) were associated with lower engagement.

**Conclusions:**

Depression-related content on TikTok is characterized by limited diagnostic completeness, regardless of creator type. Engagement appears to be driven primarily by stylistic features rather than diagnostic accuracy. These patterns raise concerns about concept creep—the gradual expansion of the psychological concept for depression—and the potential for premature self-diagnosis among young users, while also highlighting opportunities for medical professionals to adapt their communication styles on TikTok to increase both accuracy and engagement.

## Introduction

### Background

Social media platforms have become central spaces where young people are exposed to and actively search for information on mental health [1]. Among these platforms, TikTok stands out for its unparalleled reach and influence among youth, with mental health hashtags like #mentalhealth or #mentalillness amassing billions of views [2,3]. This increased accessibility to mental health information can have important benefits: It may raise awareness, reduce stigma, help young people make sense of their own experiences, foster feelings of validation and community, and encourage help-seeking among those who might otherwise feel isolated or reluctant to seek support [4]. At the same time, concerns have been raised about the accuracy of mental health content on TikTok. Much of this content is created by nonmedical professionals and is often presented using emotionally appealing stylistic features—such as background music, personal storytelling, and expressions of fear, hope, or humor [3,5-8]—which may increase relatability but also raise the risk that portrayals diverge from clinical definitions of mental illness [6,9].

Of particular concern is that mental health content characterized by diagnostic inaccuracy and appealing stylistic features tends to attract the highest levels of audience engagement [5-7,9,10]. On TikTok, engagement refers to visible indicators of user interaction, such as views, likes, comments, and shares. Importantly, such engagement metrics not only reflect a video’s popularity but also steer the platform’s recommendation system, increasing the likelihood that a video is surfaced more prominently and repeatedly within users’ feeds and distributed more widely across the platform [5]. As a result, diagnostically imprecise yet emotionally appealing mental health portrayals from nonprofessionals are likely to be disproportionately promoted across the platform, increasing their visibility and shaping young viewers’ perceptions and understanding of mental illness.

Widespread engagement with inaccurate mental health content can have several downstream consequences. It may lead to *concept creep* [11]: the gradual expansion of psychological concepts over time, broadening the accepted range of what experiences or situations encompass certain mental illnesses. When symptoms are vaguely or inaccurately portrayed, the boundaries of what constitutes a disorder may become increasingly diffuse, and the concept of that illness broadens [12]. This, in turn, can lower the threshold for *self-diagnosing*: the act of independently labeling oneself with a medical diagnosis without an official assessment by a medical professional [13]. For some individuals—particularly those facing barriers to professional care, such as financial constraints or long waiting lists—self-diagnosing may benefit their mental health. However, when diagnostic labels are applied on the basis of incomplete or inaccurate information, this may also contribute to confusion about clinical thresholds, misinterpretation of symptoms, and added pressure on already overstretched mental health systems [14].

Of particular interest in this context is depression-related content on TikTok. Not only is depression one of the most-discussed mental health issues on TikTok [2,3,7], but it is also one of the most prevalent and rising mental health disorders among young people worldwide [15]. Yet, most research on mental health content on TikTok has focused on heterogeneous mental health content, which does not permit condition-specific conclusions for depression. To the best of our knowledge, only one study has focused specifically on depression-related content on TikTok or a comparable short-form video platform; however, this study was limited to personal self-disclosure videos and did not assess the diagnostic accuracy of symptom portrayals [16]. A substantial body of research has examined depression portrayals on other social media platforms [17]—primarily text-based platforms such as Twitter (subsequently rebranded as X)—but this literature likewise has not evaluated diagnostic accuracy. This highlights the need for a comprehensive examination of depression portrayals on TikTok.

### This Study

Through a quantitative content analysis of 210 TikTok videos, this study analyzes how accurately depression symptoms are portrayed in relation to objective diagnostic criteria, what emotionally stylistic features are used to convey the content, and how these elements vary depending on whether the creator is a medical professional or not. In addition, this study explores how the diagnostic accuracy, emotionally appealing stylistic features, and creator type are associated with levels of user engagement.

### Diagnostic Accuracy of TikTok Videos About Depression

In this study, TikTok videos about depression are considered low in diagnostic accuracy when they misrepresent depression by portraying experiences that fall outside established clinical definitions—for example, labeling brief or situational sadness as “depression”—or when they rely on vague, oversimplified language (eg, “feeling down”) without providing additional clinical context. Such portrayals omit key diagnostic distinctions required to differentiate depression from everyday emotional experiences.

Little is known about the diagnostic accuracy of TikTok videos about depression and the factors determining it. Prior research on broader mental health content suggests that creator type is an important determinant of diagnostic accuracy, with videos created by medical professionals being more accurate than those created by nonmedical professionals [5,18]. We expect that this pattern also applies to depression portrayals on TikTok, leading to the following hypothesis:

- H1a: The diagnostic accuracy of TikTok videos about depression is higher when the creator is a medical professional compared to a nonprofessional.

Although videos about depression that are created by medical professionals may be higher in diagnostic accuracy, this does not necessarily translate into greater user engagement, as prior research on general mental health content on TikTok has shown that videos low in diagnostic accuracy often achieve higher levels of engagement [6,9,10]. The elaboration likelihood model (ELM) [19] offers a framework for understanding this pattern. According to the ELM, there are 2 routes of information processing: the central route, involving deliberate and thoughtful evaluation, and the peripheral route, which relies on surface-level cues, such as source attractiveness. Which processing route is taken depends largely on the individual’s motivation and ability to process the information. On TikTok, a platform designed for fast-paced, entertaining content consumption, users are more likely to process information peripherally [20]. As a result, TikTok videos that emphasize clinically detailed, high-accuracy information may receive lower engagement because they place higher cognitive demands on viewers. This leads to the following hypothesis:

- H1b: TikTok videos about depression that have lower diagnostic accuracy achieve higher engagement compared to those that have higher diagnostic accuracy.

### Personal Experiences and Emotional Appeals in TikTok Videos About Depression

Prior research on general mental health content on TikTok has shown that nonmedical professionals use more emotionally appealing stylistic features—in particular, personal experiences and emotional appeals—than medical professionals [3,5,7,8,16]. In addition, mental health videos with emotionally appealing stylistic features have consistently been linked to higher user engagement [5,7,10]. Personal experiences—often relayed with vivid emotional detail—tend to receive particularly high engagement on TikTok [2,5-7,10]. Mental health videos are also frequently emotionally charged [6] and both positive (eg, affiliation, hope, and ease [5]) and negative emotional appeals (eg, peril, fear, and status loss [5,16]) have been found to increase engagement. This is in line with identification theory, which posits that engagement is more likely when audiences psychologically and emotionally align with a media persona—such as a TikTok creator—by momentarily adopting their “identity, goals, and perspectives” [21, p. 261]. This leads to the following hypotheses:

- H2a: TikTok videos about depression will contain fewer personal experiences when the creator is a medical professional compared to a nonprofessional.
- H2b: TikTok videos about depression will contain fewer emotional appeals (positive or negative) when the creator is a medical professional compared to a nonprofessional.
- H2c: TikTok videos about depression that include personal experiences achieve higher engagement compared to those that do not include personal experiences.
- H2d: TikTok videos about depression that include emotional appeals (positive or negative) achieve higher engagement compared to those that do not include emotional appeals.

### Background Music in TikTok Videos About Depression

Background music is a third appealing stylistic feature commonly used in mental health TikTok videos [6]. As with personal experiences and emotional appeals, we expect that the use of background music in TikTok videos about depression may also differ between creator types. Where prior studies have compared the use of emotional appeals and personal experiences across creator types [5,7], no research to date has explicitly examined differences in background music use between medical professionals and nonmedical creators on TikTok. However, given their more formal communication style, medical professionals—who already tend to avoid personal narratives and emotional appeals—we expect they may likewise be less inclined to incorporate background music. To test this assumed relationship, the following hypothesis is proposed:

- H3a: TikTok videos about depression are less likely to contain background music when the creator is a medical professional compared to a nonprofessional.

It has also not been empirically tested whether background music influences engagement with mental health content. Yet, transportation theory suggests that stylistic features such as music can enhance emotional immersion into a narrative, thereby increasing engagement [22]. In addition, from the perspective of the ELM [19], background music can be interpreted as a peripheral cue that may influence engagement without requiring extensive cognitive processing. On a fast-paced platform like TikTok, where users make engagement decisions in a matter of seconds, the presence of background music may help content stand out, prompting greater user engagement. Accordingly, we hypothesize:

- H3b: TikTok videos about depression with background music achieve higher engagement compared to those without background music.

## Methods

### Ethical Considerations

This study did not involve direct interaction with human participants, as it was based on a content analysis of publicly available TikTok videos. Accordingly, informed consent and participant compensation were not applicable. The study protocol was reviewed and approved by the Ethics Review Board of the authors’ university (FMG-14125) prior to data collection. Given that the analyzed content may include identifiable information, appropriate measures were taken to ensure privacy and confidentiality. All data were collected and stored on secure university infrastructure, and no personally identifiable information is reported in this study. The study design, data collection, coding procedures, and reporting adhere to established standards for quantitative content analysis [23].

### Sample and Procedure

Using G*Power 3.1, we conducted a priori power analyses corresponding to the main analyses of the study, which indicated that a sample of 210 videos was required to detect medium effect sizes with 95% power and an α of 5%. This sample size also aligns with sample sizes of previous content analyses on general mental health portrayals on TikTok [2,5,24].

To collect our sample of videos, we relied on search term–based sampling rather than hashtag-based sampling to better approximate how users actively seek information about depression-related symptoms on TikTok. Whereas hashtag searches primarily reflect creator-driven labeling practices and platform trends—often optimized for visibility, community signaling, or reach—search queries more closely capture user-driven informational intent. To identify appropriate search terms, we conducted a pilot review designed to mimic typical user search behavior. We compared broader queries such as “depression” and “depressed” with more symptom-focused terms like “depression symptoms” and “symptoms of depression,” reviewing the first 20‐30 videos returned for each. The broader searches primarily surfaced clips that conveyed the general affective tone of depression—often through mood or music—without explicitly referencing specific symptoms. In contrast, the symptom-related queries yielded videos more directly focused on naming, describing, or illustrating depressive symptoms. Based on these findings, and in alignment with the study’s diagnostic focus, we selected 4 symptom-specific search terms that also reflect plausible user queries: “depression symptoms,” “symptoms of depression,” “depression signs,” and “signs of depression.”

To minimize preexisting algorithmic biases—such as personalized content curation based on prior user behavior, interactions, or viewing history—a new TikTok account was created for data collection. This ensured that the search results were not influenced by an established user profile and instead reflected a more neutral presentation of content. Following the approach of Jerin and colleagues [5], the web scraping tool Octoparse was used to extract an initial sampling frame of 731 TikTok videos by using the aforementioned search terms. This larger initial scrape was intended to ensure a broad enough pool of videos that would meet all inclusion criteria, allowing for the selection of the desired sample size.

**Figure 1.**
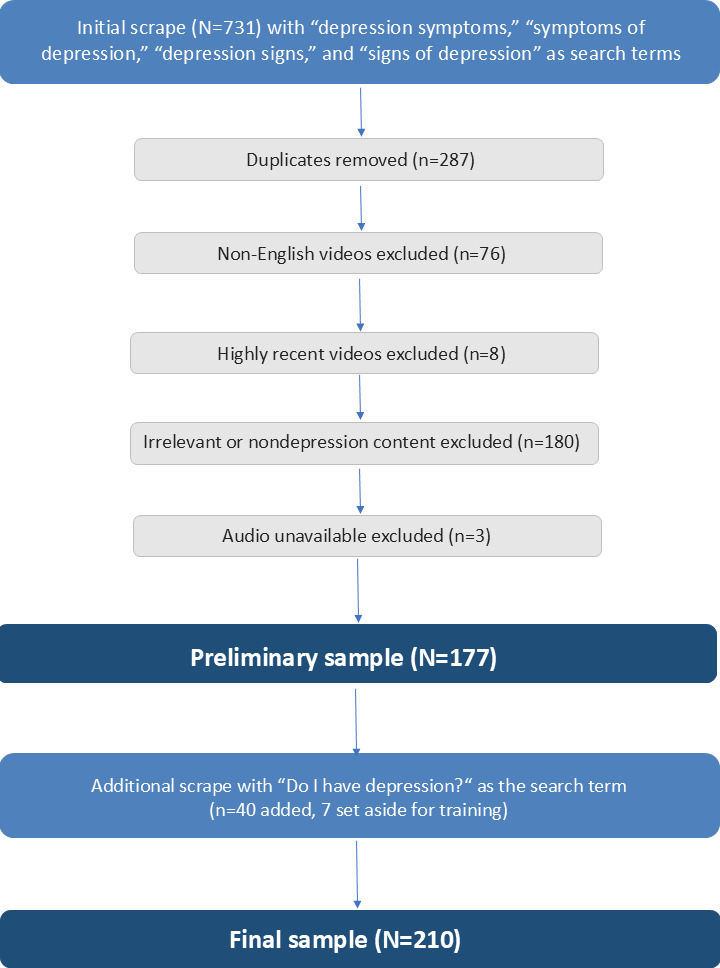
Flowchart of the TikTok video selection process, including initial scraping, exclusion steps, and the final analytic sample. “N” denotes the sample size at each stage, and “n” denotes the number of videos excluded at each step.

To ensure that only relevant and analyzable videos were included, a multistep filtering process was applied based on predefined inclusion and exclusion criteria related to language, recency, content focus, and diagnostic relevance (Figure 1). First, the sampling frame was screened to exclude duplicates (n=287) and non-English language videos (n=76), as TikTok videos had to be in English to maintain consistency in analysis and ensure clarity in interpretation. Additionally, videos posted within the last week prior to data collection were excluded (n=8) to avoid premature capture of underexposed content, since TikTok videos can continue to gain engagement several days after posting.

The remaining sampling frame of 363 was then manually filtered to include only videos that explicitly discussed or displayed at least one symptom of depression, regardless of whether the symptom was diagnostically accurate. Videos had to refer to general depression and were excluded if they focused on specific subtypes of depression (eg, postpartum depression) or addressed other mental health problems (eg, anxiety) without clearly distinguishing them from depression. In cases where the presence of depression-related symptoms was unclear, captions and hashtags were consulted; if indicators such as “#depression” were present, the video was retained under the assumption that the symptom depiction referred to depression. This filtering step resulted in the exclusion of 180 videos. Videos were included regardless of whether depressive symptoms were presented as first-person self-disclosures, secondhand descriptions of another individual’s symptoms, or general informational content.

Finally, 3 videos were excluded because the audio was not available anymore. This led to a preliminary final sample of 177. To achieve the required sample size of 210, one additional scrape using Octoparse with the search term “do I have depression” was conducted. This search phrase reflects a more personalized and diagnostic-seeking query style compared to the initial symptom-focused terms, yet still yielded content centered on symptom portrayals. After the same filtering procedure and application of exclusion criteria, this added 40 new TikTok videos to the sample, of which 7 were randomly selected to be put aside for the purpose of coder training. Consequently, through this purposive sampling method, the final sample of 210 was obtained. The sample was collected between May 5 and May 11, 2025, and all TikTok videos were either screen-recorded or downloaded (if allowed by the video’s settings) at the time of sampling.

### Coding Procedure

#### Unit of Analysis

The individual TikTok video was the unit of analysis [23], and the entire video with its caption and hashtags was considered the context frame [23]. This context frame included spoken content, on-screen overlay text, and visual depictions, as well as caption text and hashtags, but did not include user comments. A full description of the coding process, including the coding manual, can be found on the Open Science Framework [25].

#### Video Characteristics

The posting date and all engagement metrics (views, likes, comments, shares, and saves) were manually recorded at the time of sampling to capture engagement data as it appeared at the time of data collection. This procedure allowed for accurate engagement comparisons that were not affected by differences in time of coding. From this information, engagement was then calculated as the sum of likes, comments, saves, and shares (ie, active metrics that reflect a user’s deliberate decision to interact with content), while the passive metric of views was omitted. This approach follows prior work conceptualizing engagement as user‐initiated responses rather than mere exposure [7,10] and represents the outcome variable of interest for this study. During this stage, it was also recorded whether the creator self-identified as a medical professional or not by assessing explicit statements about professional status in their username, biography, caption of the video, or mentions in the video.

#### Diagnostic Accuracy

##### General Depressive Episodes

In this study, *diagnostic accuracy* was assessed through clinically recognized criteria from the *International Classification of Diseases, 11th Revision* (*ICD-11) for Mortality and Morbidity Statistics* [26]. The *ICD-11* is a globally accepted diagnostic system used by medical professionals and researchers to define and classify mental health disorders, including general depressive episodes [27]. According to the *ICD-11*, a depressive episode is defined as a period of persistently depressed mood and/or loss of interest in activities, lasting for a minimum of 2 weeks, accompanied by at least one other symptom like fatigue, feelings of worthlessness or guilt, or difficulty concentrating [26]. Hence, three *ICD-11*–based criteria were used: presence of core and associated symptoms (A), symptom frequency (B), and symptom duration (C). A fourth criterion regarding the presence of nondiagnostic symptoms was developed for the purpose of this study.

##### Criterion A: Presence of Core and Associated Symptoms

Based on the *ICD-11*, a depressive episode requires at least one of the core symptoms *depressed mood* and *diminished interest in activities*. The core symptoms must be accompanied by at least one other symptom from a list of 8 further diagnostic symptoms: *difficulty concentrating*, *feelings of worthlessness or guilt*, *hopelessness*, *recurrent thoughts of death or suicide*, *changes in sleep*, *changes in appetite, psychomotor agitation or retardation,* or *reduced energy or fatigue*. Each video was coded for the presence of these 10 symptoms. The codings were then combined to determine whether the video fulfilled this first diagnostic criterion, that is, whether it depicted at least one core symptom in conjunction with at least one additional symptom.

##### Criterion B: Symptom Frequency

Second, informed by the *ICD-11*, a depressive episode needs to be present for most of the day, nearly every day, to fulfill diagnostic criteria. This criterion was coded as 1 if it was mentioned in the video and as 0 if the mention was absent.

##### Criterion C: Symptom Duration

The third criterion of the *ICD-11* is the duration of the symptoms, as they must be present for at least 2 weeks. This duration criterion was again coded as 1 for present and 0 for absent.

##### Criterion D: Mention of Nondiagnostic Symptoms

This study-specific criterion assessed whether the video included portrayals of symptoms not recognized by the *ICD-11*. In our initial exploratory screening process, we cataloged the most common and recurring non–*ICD-11* symptoms mentioned and constructed a predefined list of 17 nondiagnostic symptoms. An “other” category was provided, allowing coders to note any additional symptoms not covered by the predetermined list. Examples of nondiagnostic symptoms include “putting on a front” or “messy room.” Nondiagnostic symptoms were coded as present (=1) or absent (=0); if no nondiagnostic symptom was present in a video, it was later recoded as 1 for the purposes of the diagnostic accuracy score.

To calculate a diagnostic accuracy score for every video, the 4 criteria (A-D) were summed to create a composite score ranging from 0 to 4, with higher values reflecting a more accurate diagnostic depiction of depression. Because the presence of nondiagnostic symptoms indicates inaccuracy, their absence contributed positively to the overall score.

### Personal Experiences, Emotional Appeals, and Background Music

Each video was coded for 3 stylistic features: personal experiences, emotional appeals, and background music. Personal experiences were coded as present when the video included personal storytelling elements (eg, the creator’s own experience or that of someone they know). Emotional appeals were coded as present when the video conveyed emotional expressions, categorized as negative (eg, sadness, fear, shame), humorous (eg, sarcasm, irony), or positive (eg, hope, encouragement, empowerment). Background music was coded for its presence or absence, defined as any musical track or sound layered beneath spoken or visual content, and its valence was classified as sad, neutral, or uplifting.

### Control Variables

Following prior TikTok content analyses [3,6,8], we coded for presentation style to account for basic differences in how videos are delivered, which may independently shape engagement. Presentation style categories were adopted from previous work [3,8] and slightly modified to better fit the corpus under study. Each video was coded for its dominant presentation style, categorized into “speaking directly to camera,” “acted-out scenes,” “animation,” “movie or TV scene,” “text-centered videos,” “someone giving a speech,” “podcast interview,” or an open “other” category for formats not captured by these options.

### Reliability Testing

For intercoder reliability (ICR) testing of the coding scheme, an independent coder was trained by the first author, who then participated in the coding process. A set of 7 videos was used for coder training, which were not part of the final dataset. The training began with an introduction session in which 2 TikTok videos were used as examples to explain the coding process. The second coder then independently coded 3 TikTok videos and received detailed feedback. Any uncertainties were discussed, and the coding scheme was refined accordingly. Finally, 2 TikTok videos were used for a concluding training session, after which no further questions remained.

Following the training phase, each coder coded a randomly selected 10% of the sample (n=21), a threshold that adheres to existing guidelines to calculate ICR [23]. Krippendorff α was used as the reliability coefficient due to its commonly argued superiority for applicability to relatively small sample sizes and all levels of data, including dichotomous variables [23,28,29]. Most variables demonstrated good levels of reliability, ranging from a moderate α of 0.74 to exceeding a strong α of ≥0.8 [30]. Six variables were below the accepted minimum threshold of α≥0.67 [30]. Two of these were adjusted (“negative emotional appeal” was combined with the other two types of emotional appeals into a new variable “emotional appeal”) or removed (“background music valence”). For the remaining 4 with an α of 0.65 (“presentation style: other,” “nondiagnostic symptom: physical signs,” “mention of daily occurrence,” and “emotional appeal”), agreement among coders was 95%. Because of this high level of coder agreement but low Krippendorff α levels, Gwet AC1 (agreement coefficient 1) was calculated as an additional reliability coefficient to estimate expected agreement for these variables, as suggested by Riffe and colleagues [23]. All variables reached Gwet AC1 of 0.95, indicating excellent reliability. Consequently, these variables were retained. Table A1 in Open Science Framework [25] details all reliability statistics.

### Statistical Analyses

To examine whether TikTok videos created by medical professionals scored higher on diagnostic accuracy than those created by nonprofessionals (H1a), a nonparametric Mann-Whitney *U* test was conducted with creator type as the independent variable and the diagnostic accuracy score (ranging from 0 to 4) as the dependent variable. A nonparametric test was chosen instead of a *t* test because Shapiro-Wilk tests revealed that the diagnostic accuracy score was not normally distributed (medical professionals: *W*(72)=0.886, *P*<.001; nonprofessionals: *W*(138)=0.857, *P*<.001). Since the a priori power analysis was based on a *t* test, a post hoc power analysis using G*Power 3.1 was conducted for nonparametric testing, revealing 99.2% of power to detect medium effects (α=0.05, *w*=0.30, n=210; [31]). Because Mann-Whitney *U* tests compare the distribution of ranks rather than means, results are reported in terms of the median diagnostic accuracy score.

To test whether a lower diagnostic accuracy score predicted higher user engagement (H1b), a hierarchical regression analysis was conducted. The independent variable was the diagnostic accuracy score, and the dependent variable was the engagement score. The engagement score was log-transformed to correct extreme positive skew and reduce the influence of outliers, a recommended practice for highly skewed data [32]. All assumptions for hierarchical regression (normality, homoscedasticity, and absence of multicollinearity) were checked and met. In the first step, we entered the control variables creator type (medical professional vs nonprofessional) and the 4 most common presentation styles (direct-to-camera formats, text-based videos, acted-out scenes, and animations). In the second step, the diagnostic accuracy score was added to the model to examine its unique association with engagement above and beyond these controls.

To assess whether TikTok videos created by medical professionals feature fewer personal experiences (H2a), emotional appeals (H2b), and background music (H3a) than those created by nonprofessionals, 3 chi-square tests of independence were conducted. To examine whether the presence of personal experiences (H2c), emotional appeals (H2d), and background music (H3b) leads to higher engagement, 3 hierarchical regression analyses were conducted. The independent variables were the presence (vs absence) of (1) personal experiences, (2) emotional appeals, and (3) background music. The dependent variable was the log-transformed engagement score. Creator type and presentation styles were again entered as control variables as a first step, followed by the focal stylistic predictor in the second step.

## Results

### Descriptive Statistics

#### Depression Symptom Portrayals

Each of the 210 TikTok videos was coded for the presence of diagnostic and nondiagnostic depression symptoms. The most common diagnostic symptoms were “depressed mood” (n=131, 62.4%), “reduced energy or fatigue” (n=82, 39%), and “changes in sleep” (n=66, 31.4%). Nondiagnostic symptoms—those not included in the *ICD-11*—were also common, with 134 (63.8%) videos referencing at least one. The 3 most mentioned nondiagnostic symptoms were “putting on a front” (n=45, 21.4%), “social isolation” (n=30, 14.3%), and “irritability” (n=22, 10.5%). Distributions of diagnostic and nondiagnostic symptoms are presented in Tables1  2, respectively.

**Table 1.** Distribution of *ICD-11*^a^ diagnostic symptoms in TikTok videos about depression (N=210)^b^.

*ICD-11* symptoms	Videos, n (%)
Depressed mood	131 (62.4)
Reduced energy or fatigue	82 (39.0)
Changes in sleep	66 (31.4)
Hopelessness	61 (29.0)
Feelings of worthlessness	58 (27.6)
Changes in appetite	53 (25.2)
Loss of interest	53 (25.2)
Difficulty concentrating	37 (17.6)
Suicidal thoughts	33 (15.7)
Psychomotor agitation	11 (5.2)

a *ICD-11: International Classification of Diseases, 11th Revision*.

b The table shows the number and proportion of TikTok videos in which each *ICD-11* diagnostic symptom of depression was portrayed, based on a content analysis of English-language TikTok videos.

**Table 2.** Distribution of nondiagnostic symptoms in TikTok videos about depression (N=210)^a^.

Symptom	Videos, n (%)
Putting on a front	45 (21.4)
(Social) isolation	30 (14.3)
Irritability	22 (10.5)
Lack of self-care	19 (9.0)
Loneliness	19 (9.0)
Media distractions	14 (6.7)
Low motivation	13 (6.2)
Body pains	13 (6.2)
Drug (ab)use	12 (5.7)
Messy room	11 (5.2)

a The table shows the number and proportion of TikTok videos in which each nondiagnostic symptom (ie, symptoms not recognized in *International Classification of Diseases, 11th Revision* (*ICD-11*) criteria for depressive episodes) was portrayed, based on a content analysis of English-language TikTok videos. The table lists the 10 most prevalent nondiagnostic symptoms; other nondiagnostic symptoms coded for occurred in <5% of videos.

#### Diagnostic Accuracy of TikTok Videos About Depression

Each video was evaluated against 4 diagnostic accuracy criteria. To meet the first criterion, a video had to depict at least one core depression symptom and at least one additional symptom. In total, 111 (52.9%) TikTok videos met this criterion. The second criterion, referring to the daily occurrence of symptoms, was met in 42 (20%) videos. The third criterion, requiring symptoms to persist for at least 2 weeks, held for 25 (11.9%) videos. The fourth criterion, absence of nondiagnostic symptom mentions, was met in 76 (36.2%) videos. Overall, 59 (28.1%) videos met none of the criteria, 78 (37.1%) met exactly 1 criterion, 50 (23.8%) met 2 criteria, 16 (7.6%) met 3 criteria, and only 7 (3.3%) met all 4 criteria. The total diagnostic accuracy score ranged from 0 to 4 with a mean of 1.21 (SD 1.04), indicating that, on average, videos met just over one diagnostic criterion.

#### Creator and Stylistic Features of Depression-Related TikTok Videos

In terms of creator type, 138 (65.7%) TikTok videos were posted by creators without any indication of a professional medical background, while 72 (34.3%) were created by medical professionals. With respect to the key emotional stylistic features examined, 174 (82.9%) videos included background music, 164 (78.1%) featured emotional appeals, and 80 (38.1%) included personal experiences. The most common presentation style was in the form of creators speaking directly to the camera (n=66, 31.4%), followed by text-based presentations (n=62, 29.5%), acted-out scenes (n=41, 19.5%), and animation (n=19, 9%).

#### Engagement Metrics of TikTok Videos About Depression

All individual and total engagement metrics are visualized in Table 3. On average, the total engagement score of a TikTok video about depression in our sample was 260,880 (SD 609,338). All engagement variables showed substantial positive skewness, indicating that a few outliers achieved disproportionately high numbers of engagement.

**Table 3.** Engagement metrics of TikTok videos about depression (N=210)^a^.

	Descriptive statistics			
Engagement metrics	Min	Max	Mean (SD)	Skewness
Likes	2	4,200,000	220,431 (500,476)	4.19
Comments	0	31,900	1726 (3941)	4.62
Saves	0	967,100	29,556 (88,337)	7.36
Shares	0	430,500	9167 (33,978)	9.79
Engagement	2	5,622,300	260,880 (609,338)	4.84

a Descriptive statistics are based on a content analysis of English-language TikTok videos about depression. Engagement is operationalized as the sum of likes, comments, saves, and shares. Skewness indicates the asymmetry of the distribution of each engagement metric. A skewness value above 1 suggests a highly right-skewed distribution.

### Hypothesis Testing

#### H1a: Creator Type and Diagnostic Accuracy

The median diagnostic accuracy score for medical professionals was higher (median 1.40, IQR 1-2) than for nonprofessionals (median 1.11, IQR 0-2); however, the difference was not statistically significant (*U*=4208, *z*=−1.90; *P*=.06). Given the overall low diagnostic accuracy score across the TikTok videos (mean 1.21, SD 1.04), an exploratory analysis was conducted to examine whether medical professionals (n=72) and nonprofessionals (n=138) significantly differed in how they fulfilled each of the 4 individual diagnostic criteria. The first criterion—mentioning at least one core and one additional symptom—was met in 49 (68.1%) videos created by medical professionals, and in 62 (44.9%) videos created by nonprofessionals, revealing a significant difference (*U*=3819; *z*=−3.18; *P*=.001). The second criterion, mentioning daily occurrence of symptoms, was mentioned in 14 (19.4%) professional and 28 (20.3%) nonprofessional videos, revealing no significant difference (*U*=4926; *z*=−0.15; *P*=.89). The third criterion—mentioning symptom duration—was proportionally slightly more often fulfilled by medical professionals (n=12, 16.7%) than by nonprofessionals (n=13, 9.4%), but this difference was not significant (*U*=4608; *z*=−1.54; *P*=.13). Lastly, the fourth criterion, not mentioning nondiagnostic symptoms, was met in 26 (36.1%) medical professional videos and in 50 (36.2%) nonprofessional videos (*U*=4962; *z*=−0.02; *P*=.99).

#### H1b: Influence of Diagnostic Accuracy on Engagement

To examine whether diagnostic accuracy predicted engagement, we conducted a hierarchical linear regression analysis. The first model—with only the control variables creator type and presentation style—was statistically significant (*F*_5,204_=4.04; *P*=.002), explaining 9% of the variance in engagement (*R*^2^=0.09). Adding diagnostic accuracy in the second model did not significantly improve model fit (Δ*R*^2^=0.008; Δ*F*_1,203_=1.77; *P*=.19), and diagnostic accuracy was not a significant predictor of engagement (*B*=−0.10, SE 0.08; *P*=.19). However, 2 control variables were associated with significantly lower engagement: videos featuring creators speaking directly to the camera (*B*=−0.62, SE 0.23; *P*=.008) and videos centered around text (*B*=−0.65, SE 0.21; *P*=.003). This highlights that presentation style is a distinct factor influencing engagement.

#### H2a, H2b, and H3a: Creator Type and Use of Stylistic Features

The use of personal experiences differed by creator type (*χ*²_1_[n=210]=41.15, *P*<.001). TikTok videos created by medical professionals contained significantly fewer personal experiences (n=6, 8.3%) compared to nonprofessionals (n=74, 53.6%). The difference was moderate in strength (*φ*=0.44) [31]. The use of emotional appeals also differed across creator types (*χ*²_1_[n=210]=25.01, *P*<.001). Emotional appeals appeared in 42 (58.3%) TikTok videos created by medical professionals and in 122 (88.4%) nonprofessional TikTok videos, another moderate difference (*φ*=0.35) [31]. Finally, the third chi-square revealed a significant difference in the use of background music (*χ*²_1_[n=210]=11.15, *P*<.001), with a small effect size (*φ*=0.23) [31]. Background music was less often used in medical professional TikTok videos (n=51, 70.8%) compared to nonprofessional TikTok videos (n=123, 89.1%).

#### H2c, H2d, H3b: Influence of Stylistic Features on Engagement

To test whether personal experiences (H2c), emotional appeals (H2d), or background music (H3b) were associated with engagement, 3 hierarchical regression analyses were conducted. Adding personal experiences to the model resulted in a significant increase in explained variance (Δ*R*²=0.024, *P*=.02), indicating that videos with personal experiences achieved higher engagement (*B*=0.41, SE 0.18; *P*=.02). Similarly, adding emotional appeals significantly improved the model fit (Δ*R*²=0.059, *P*<.001), also positively predicting engagement (*B*=0.73, SE 0.19, *P*<.001). Finally, the inclusion of background music also resulted in a significant increase in explained variance (Δ*R*²=0.027, *P*=.01), with background music positively predicting engagement (*B*=0.54, SE 0.22; *P*=.01). Across all models, videos featuring creators speaking directly to the camera (*B*s ranging from −0.49 to −0.69; .003≤*P*≤.04) and videos centered around text (*B*s ranging from −0.56 to −0.64; .002≤*P*<.01) consistently predicted lower engagement.

## Discussion

### Summary

This study analyzed 210 TikTok videos about depression and found that, overall, diagnostic accuracy was low, with no significant differences between content created by medical professionals and nonprofessionals. Lower diagnostic accuracy itself was not associated with higher engagement. Rather, videos featuring personal experiences, emotional appeals, and background music—stylistic elements more commonly found in nonprofessional content—were found to be the drivers behind higher engagement. By contrast, more static formats such as direct-to-camera explanations and text-centered videos were associated with lower engagement. Altogether, these findings indicate that engagement with depression-related TikTok videos is dependent on stylistic elements and emotional appeals, rather than diagnostic accuracy.

### Diagnostic Accuracy of Depression Videos on TikTok

Contrary to our hypothesis, lower diagnostic accuracy was not a significant predictor of higher engagement, challenging prior research on mental health content on TikTok [6,9,10]. While this at first presents a promising finding, it is important to highlight that the diagnostic accuracy was low across the entire sample. Specifically, although around half of the TikTok videos met the first diagnostic accuracy criterion by mentioning at least one core and one additional diagnostic symptom, the other 3 criteria—referencing daily occurrence, a duration of at least 2 weeks, and avoiding nondiagnostic symptoms—were rarely fulfilled. Importantly, diagnostic accuracy in this study reflects alignment with established clinical diagnostic criteria rather than an evaluation of the authenticity or validity of lived experiences shared on the platform. As such, low diagnostic accuracy should be interpreted as limited diagnostic completeness, not as an invalidation of personal experiences with distress or depression.

At the same time, limited diagnostic completeness may contribute to concept creep [11], whereby the meaning of clinical terms such as “depression” gradually expands to encompass a broader range of experiences. When diagnostic boundaries become diffuse, this may increase the likelihood of self-diagnosing based on incomplete information. Moreover, an experimental study in the context of attention-deficit/hyperactivity disorder (ADHD) misinformation on TikTok found that misinformed emerging adults had higher confidence in their ADHD knowledge and higher treatment-seeking intentions than emerging adults who were not exposed to such misinformation [33]. This highlights that inaccurate content can create a false sense of understanding and may add pressure on already overstretched mental health services. It is crucial for future research to assess how exposure to varying levels of diagnostic accuracy in depression portrayals on TikTok affects self-diagnosing behaviors and concept creep.

### Diagnostic Accuracy of Medical Professionals vs Nonprofessional Creators

Strikingly, the low diagnostic accuracy also characterized depression portrayals by medical professionals, whose videos were not significantly more accurate than those of nonprofessionals. One possible explanation for this finding is that TikTok’s fast-paced and short-form video format encourages even professionals to simplify their content and incorporate nondiagnostic, more relatable symptom portrayals. In doing so, they may prioritize accessibility over diagnostic depth, omitting key criteria for clarity or conciseness. At the same time, however, our findings show that medical professionals relied less on implementing personal experiences, emotional appeals, and background music in their content compared to nonprofessionals. This aligns with previous findings in the mental health space [5,7] and suggests that medical professionals may simplify *what* they communicate without fully adapting *how* they communicate to the platform’s engagement norms. Future research should specifically examine why so many medical professionals choose to add nonclinical symptom portrayals and omit essential *ICD-11* criteria when adapting their messages for TikTok, and how they balance clinical responsibility with platform-specific communication norms. In-depth interviews or focus groups with medical professionals can add to a better understanding of how they adapt to the platform.

While the fact that medical professionals did not produce more diagnostically accurate content may be understandable in light of their efforts to reduce cognitive load, it raises serious concerns from the perspective of the ELM of persuasion. Medical professionals hold source credibility, a powerful peripheral cue in the ELM that can have a strong influence on persuasion and trust [34]. As such, the disconnect between diagnostic accuracy and medical professional status found in this study is problematic, as users may be persuaded by the authority of the source, even when the message lacks diagnostic accuracy. In a platform environment where users rely heavily on heuristics [20], the persuasive power of credibility without accuracy may contribute to the spread of misleading or oversimplified information.

### How Stylistic Features Drive Engagement

Emotional appeals, personal experiences, and background music were all common features of the TikTok videos in our sample. In line with our hypotheses, the presence of each of these features individually predicted significantly higher engagement, supporting earlier studies about mental health content on TikTok [2,5-7,10]. These findings are also in line with identification theory [21], which suggests that users may become more engaged when they can emotionally and psychologically align with a media persona.

This study’s findings further align with notions from transportation theory [22]. Not only did the presence of background music increase engagement, but among the 4 most common presentation styles that were tested across our analyses, direct-to-camera and text-based formats consistently led to lower engagement. This suggests that purely informational or static formats may not be sufficient to capture users’ attention on a highly dynamic platform like TikTok, regardless of whether the creator is a medical professional or not. Future research could explore these dynamics more directly by using experimental designs to assess whether variations in background music and presentation style influence users’ sense of transportation and therefore engagement with the content.

While diagnostic accuracy did not predict engagement patterns and was low overall, it remains unclear whether higher diagnostic accuracy would undermine or enhance the effectiveness of stylistic features. Importantly, the danger of heightened identification and transportation lies in the possibility that users may connect more deeply with diagnostically inaccurate content. However, as young people increasingly rely on online resources and information as part of their mental health help-seeking process [35], high diagnostic accuracy of such resources could serve a more constructive role and act as a stepping stone toward seeking offline help [36]. In this context, TikTok’s engaging and accessible format positions it as a particularly promising platform for mental health communication [37]. Future experimental research should examine whether stylistic features still lead to increased engagement when combined with accurate symptom portrayals of depression.

### Practical Implications

The majority of TikTok videos about depression in our sample were posted by creators without any indication of a professional medical background and showed very low diagnostic accuracy. Although such videos may not be intended to provide diagnostic guidance, they are frequently encountered by users seeking information about depression and may shape how viewers interpret their own experiences, draw conclusions about whether their symptoms “count” as depression, and influence help-seeking decisions. This underscores the need for clearer contextual cues to support the interpretation of mental health–related content. Platforms such as TikTok could consider introducing labels or informational prompts that clarify whether content reflects professional medical information and direct users to authoritative resources. Such measures could help reduce misinterpretation without restricting creators’ ability to share lived experiences.

However, directing users toward professional content is only beneficial if such content is diagnostically accurate. Yet, in our sample, medical professionals frequently mentioned nondiagnostic symptoms and omitted the daily occurrence criterion at rates similar to nonprofessionals. To improve the quality of their TikTok content, medical professionals should avoid vague symptom portrayals and place greater emphasis on clearly communicating key clinical criteria. At the same time, medical professionals can enhance engagement by incorporating stylistic features such as background music, emotionally appealing framing, or patient-informed narratives that include personal experiences, all without compromising clinical integrity. Importantly, they should also avoid overly static formats and information-only presentation styles, as these were associated with lower engagement. Future research should experimentally test how medical professional content that integrates both high diagnostic accuracy and stylistically engaging features performs in terms of engagement on TikTok. Such work could guide the development of evidence-based best practices for professional mental health communication on TikTok.

### Limitations

Although this study offers novel insights into the diagnostic accuracy and stylistic features of TikTok videos about depression by developing an objective and replicable diagnostic coding tool for evaluating depression symptom portrayals, several limitations should be noted. First, regarding the sample, while the included videos were posted between February 2020 and April 2025, most dated to the period between 2022 and 2024. As TikTok’s platform affordances, content norms, and mental health discourse evolve rapidly, the findings should be interpreted in light of this temporal context. In addition, the sample size was necessarily limited given the labor-intensive nature of manual content analysis. Future research could build on the diagnostic accuracy coding framework developed in this study by applying automated content analysis approaches to examine depression portrayals at a much larger scale.

Second, the *ICD-11* criteria may have been overly strict given the nature of TikTok as an entertainment-oriented platform. Creators, whether medical professionals or not, often aim to simplify complex topics to fit the platform’s short-form video format, thereby omitting certain *ICD-11* criteria. A more flexible or scaled application of *ICD-11* criteria might better capture how diagnostic elements are adapted for such contexts in future studies. Third, while the inclusion of more detailed coding in terms of valence (ie, distinguishing between a positive and negative emotional appeal, coding the emotional valence of the background music) was intended, low ICR prevented its implementation. Future research should leverage automated content‐analysis methods, such as audio valence detection models that can reliably classify the emotional tone of background music, and natural language processing algorithms to more precisely identify and quantify specific emotional appeals.

Lastly, engagement was measured only in terms of active interactions (likes, comments, shares, and saves), not passive reach (views). Although reach data was available and could have been added to the engagement score, it was excluded because, in line with prior research, engagement was conceptualized as a form of user interaction that reflects an active decision to respond to content, rather than mere exposure [7,10]. Nevertheless, including reach metrics in future research could offer a more comprehensive understanding of content visibility and its potential influence.

### Conclusions

Our study shows that depression content on TikTok is often low in diagnostic accuracy, both among medical professional creators and nonprofessional creators. Although this low diagnostic accuracy itself did not significantly drive engagement, stylistic features did: the presence of personal experiences, emotional appeals, and background music was each associated with higher engagement. By contrast, we found that videos from medical professionals and more static, information-heavy formats drew less engagement. These patterns raise concerns about concept creep and potential premature self-diagnosis, but they also point to a clear opportunity: clinically accurate information can be made more compelling if professionals adopt platform-native content styles. Practically, this means foregrounding clear symptom criteria alongside human-centered, emotionally resonant presentation and sensory cues. Future research has the opportunity to develop evidence-based best practices for professional mental health communication on short-form video platforms. TikTok, if used thoughtfully, can be part of—and not a barrier to—credible, engaging depression education for young audiences.

## Abbreviations

AC1: agreement coefficient 1
ADHD: attention-deficit/hyperactivity disorder
ELM: elaboration likelihood model
ICD-11: International Classification of Diseases, 11th Revision
ICR: intercoder reliability
